# Spine Surgery with Electronic Conductivity Device: A Prospectively Multicenter Randomized Clinical Trial and Literature Review

**DOI:** 10.1111/os.13880

**Published:** 2023-09-22

**Authors:** Xiao Zhai, Bo Li, Kai Chen, Ziqiang Chen, Jie Shao, Kai Chen, Qintong Xu, Dehua Meng, Qinming Fei, Leisheng Jiang, Yushu Bai, Ming Li

**Affiliations:** ^1^ Department of Orthopaedics, Shanghai Changhai Hospital Navy Medical University Shanghai China; ^2^ Department of Orthopaedic Surgery, Zhongshan Hospital Fudan University Shanghai China; ^3^ Spine Center, Xinhua Hospital Shanghai Jiaotong University School of Medicine Shanghai China

**Keywords:** Accuracy, Electronic conductivity device, Navigation surgery, Pedicle screw, Radiation exposure

## Abstract

**Objective:**

Improving accuracy and safety of pedicle screw placement is of great clinical importance. Electronic conductivity device (ECD) can be a promising technique with features of affordability, portability, and real‐time detection capabilities. This study aimed to validate the safety and effectiveness of a modified ECD.

**Methods:**

The ECD underwent a modification where six lamps of various colors, and it was utilized in a prospectively multicenter randomized controlled clinical trial involving 96 patients across three hospitals from June 2018 to December 2018. The trial incorporated a self‐control randomization with an equal distribution of left or right side of vertebral pedicle among two groups: the free‐hand group and the ECD group. A total of 496 pedicle screws were inserted, with 248 inserted in each group. The primary outcomes focused on the accuracy of pedicle screw placement and the frequency of intraoperative X‐rays. Meanwhile, the secondary indicator measured the time required for pedicle screw placement. Results were presented as means ± SD. Paired samples *t*‐test and *χ* ^2^‐test were used for comparison. Furthermore, an updated review was conducted, which included studies published from 2006 onwards.

**Results:**

Baseline patient characteristics were recorded. The primary accuracy outcome revealed a 96.77% accuracy rate in the ECD group, compared to a 95.16% accuracy rate in the free‐hand group, with no significant differences noted. In contrast, ECD demonstrated a significant reduction in radiation exposure frequency when compared to the free‐hand group (1.11 ± 0.32 vs. 1.30 ± 0.53; *p* < 0.001), resulting in a 14.6% reduction. Moreover, ECD displayed a decrease of 30.38% in insertion time (70.88 ± 30.51 vs. 101.82 ± 54.00 s; *p* < 0.001). According to the results of the 21 studies, ECD has been utilized in various areas of the spine such as the atlas, thoracic and lumbar spine, as well as sacral 2‐alar‐iliac. The accuracy of ECD ranged from 85% to 100%.

**Conclusion:**

The prospectively randomized trial and the review indicate that the use of ECD presents a secure and precise approach to the placement of pedicle screws, with the added benefit of reducing both procedure time and radiation exposure.

## Graphical abstract

The electronic conductivity device (ECD), which operates on the principles of bioelectric impedance to provide real‐time intraoperative feedback during freehand drilling utilizing lights and sounds.

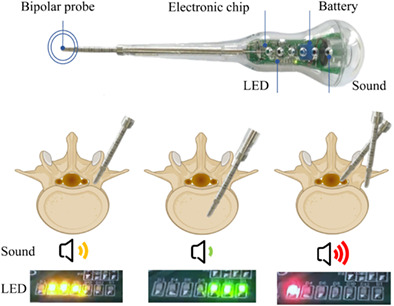

## Introduction

The posterior pedicle screw system has gained widespread use in various spine surgeries for the treatment of lumbar disc herniation (LDH), lumbar spinal stenosis (LSS), degenerative spinal deformity, spondylolisthesis, and vertebral compression fracture.^1^ Misplacement of pedicle screws is a recurring issue, which poses a significant risk of complications due to its proximity to the spinal canal and surrounding vessels.^2^ Studies have indicated that the misplacement rate can be as high as 5%–39% during pedicle screw insertion, especially in scoliosis patients.^3^, ^4^, ^5^ Surgeons’ experience is a crucial determinant of accuracy, with less experienced spine surgeons being associated with significantly more pedicle screw breaches compared to their more experienced counterparts.^6^, ^7^ Therefore, improving the accuracy and safety of pedicle screw placement is paramount to mitigate potential risks and related problems.

Several methodologies have been developed to manage the potential risks associated with screw misplacement, particularly during pedicle screw placement procedures.^8^ One such approach, computer‐assisted surgery (CAS), has been shown to enhance accuracy, albeit with some complexity and time requirements.^9^ Another option, augmented reality (AR) navigation, allows anatomical visualization to enhance surgical workflow, but is limited in its ability to provide direct feedback.^10^, ^11^ Recently benefits such as preoperative planning, high accuracy of screw insertion, and low radiation exposure have made the use of robot‐assisted systems increasingly popular,^12^ but it is expensive and hard to equip in developing areas. To optimize navigation systems for pedicle screw placement, ideal qualities that would be beneficial include ease of use, affordability, portability, and real‐time detection capabilities.

One promising solution has been the introduction of the electronic conductivity device (ECD),^13^ which operates on the principles of bioelectric impedance to provide real‐time intraoperative feedback during freehand drilling utilizing lights and sounds.^14^ The ECD does not require preoperative imaging or any form of ionizing radiation. The initial report on detecting iatrogenic initial pedicle perforation in pigs using the ECD was made by Bolger *et al*.^13^ Since then, this technique has been tested in animal, cadaver, and clinical trials.^15^, ^16^, ^17^, ^18^ Although the results have been promising, one limitation of the ECD is that it only uses a single monochromatic signal light to determine the magnitude of resistance impedance. This lack of a visual signal similar to a traffic light may make it less user‐friendly. Moreover, the patients included in the mentioned studies were divided into different groups based on the use of ECD. However, there were notable variations in bioelectric impedance among patients with different ages and weights. Consequently, it would be more appropriate to plan a multi‐center prospective randomized control trial utilizing self‐control randomization techniques in order to account for potential biases related to patients and vertebral factors.

**FIGURE 1:**
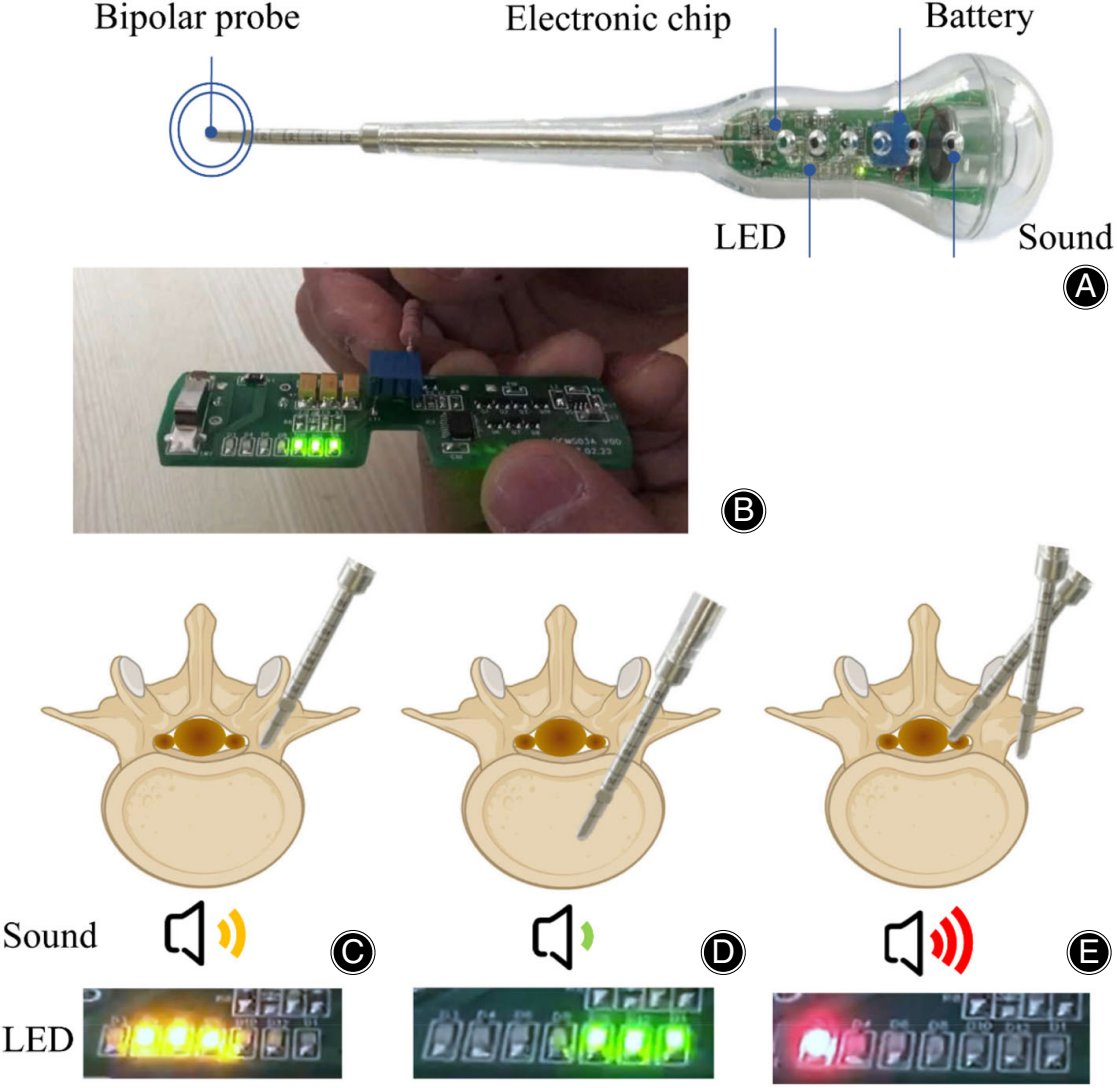
Electronic conductivity device (ECD) and operating principle. (A, B) A sample of ECD with lights and sounds for alarming. (C) When the tip of ECD touches the cortical bone, the frequency of the sound is moderate and the light is yellow. (D) When the tip of ECD drills into the cancellous bone, it sounds gentle and shows green light. (E) When the tip of ECD contacts with soft tissue or blood outside of the cortical bone, it emits sounds at a high frequency and lights of red as warning signals.

The purpose of the study is as following: (i) to improve the ECD by incorporating changes in the colors and quantities of lights to provide informative cues, we have made alterations to the ECD wherein six lamps of diverse colors have been incorporated on the progress bar (Figure 1A,B). Ascertaining the safety aspects, it can be concluded that red lights indicate soft tissue or blood warning, green lights are indicative of safe trajectory direction, and yellow light necessitates patient attention for adjustment as the tip may encounter cortical bone (Figure 1C–E); (ii) to validate the safety and effectiveness of the modified ECD, a prospectively multicenter randomized control clinical study was undertaken, and a self‐control randomization was designed to minimize the bias among patients; and (iii) to elucidate the research progress of this device, an updated review was conducted.

## Materials and Methods

The study was a prospectively multicenter randomized control clinical trial that took place in three grade A tertiary hospitals in Shanghai, China. It obtained institutional ethical committee approval and written patient consent prior to commencing (CHEC2017‐179, Shanghai Changhai Hospital Ethics Committee; 2017‐101, Shanghai Zhongshan Hospital Ethics Committee; HXEC‐B‐2017‐004‐2, Ethics Committee of Xinhua Hospital). The clinical trial was registered with the Chinese Clinical Trial Registry under the identifier ChiCTR1800016744, and remained unchanged from start to finish.

### Modification of ECD

We developed a modified ECD (Shanghai Jiguang Medical Technology Co., Ltd., Shanghai, China, Patent No: 201821826836.7) that features a progress bar comprising six differently colored lamps. At the tip of the ECD, a bipolar probe measures tissue conductivities at a rate of five times per second before converting the signals into lights and sounds. Specifically, upon touching the cortical bone, the sound frequency is moderate and the light is yellow. As the ECD moves into the cancellous bone, the sound becomes gentler and the light changes to green. If soft tissue or blood is encountered outside of the cortical bone, the ECD emits sounds at a high frequency and displays a red light to warn users (see Video S1).

### Patients

From June 2018 to December 2018, all consecutive patients suffering from spinal diseases were prospectively enrolled treated by YB, SJ, and QF.

Inclusion criteria were as following: (i) aged 18–80 years, male, or female; (ii) first time undergoing spinal fixation surgery, and the pedicle screw implanted within levels from T1 to S1; and (iii) able to understand and participate in this clinical trial voluntarily.

Exclusion criteria were as following: (i) the operating segments of the vertebrae have pathological changes (such as tumors and fractures), which might cause significant changes in the electrical impedance; (ii) pregnant and lactating women; (iii) patients who have undergone spinal fusion; (iv) participation in other clinical trials at the same time; (v) other navigation methods are used during the surgery; and (vi) patients with heart pacemakers or other active medical equipment.

The study enrolled a total of 96 patients who were subjected to a self‐control randomization design. The left or right side was allocated to two groups, namely the free‐hand group and the ECD group, in a 1:1 ratio. The primary aim of this allocation was to maintain a standardized vertebral micro‐environment between the two groups. A computer‐generated random side was conducted: if the left side was defined to be performed by the free‐hand technique, the right side was defined to use ECD, and *vice versa*. To minimize the bias that the surgeon may be easier to find the second side, the left side was defined to be started first.^19^ These measures were undertaken to seek minimum bias during the study.

### Interventions

All three of the surgeons possess more than 20 years of experience in spine surgeries. They were scheduled to learn the manual of ECD and the protocol of the clinical trial in the project launch meeting. No training (on pigs) was provided since ECD does not change the flow of the operation and the habits of surgeons. The assigned posterior lumbar interbody fusion (PLIF), transforaminal lumbar interbody fusion (TLIF), or lateral interbody fusion (LIF), was conducted in an unblinded manner. General anesthesia was administered, and patients were placed in a prone position on the Jackson table with an empty abdomen. Following routine disinfection and draping, a skin incision was made, and paravertebral muscle dissection was performed. The vertebral pedicles were drilled using a free‐hand technique or ECD, and pedicle screws were subsequently inserted. Intraoperative confirmation of the position of the pedicle screws was achieved using C‐arm fluoroscopy, with anteroposterior and lateral images captured by a technician. The screws were adjusted as necessary and re‐examined using C‐arm fluoroscopy. The surgeons performed decompression, and then secured the rod.

All patients received postoperative auxiliary medicine, CT, and X‐ray examination, and were discharged without problems in 3–5 days.

### Data Collection and Analysis

We collected data in the hospital including: age, sex, height, weight, smoking, drinking alcohol, primary reason for procedure, and fusion levels.

**FIGURE 2:**
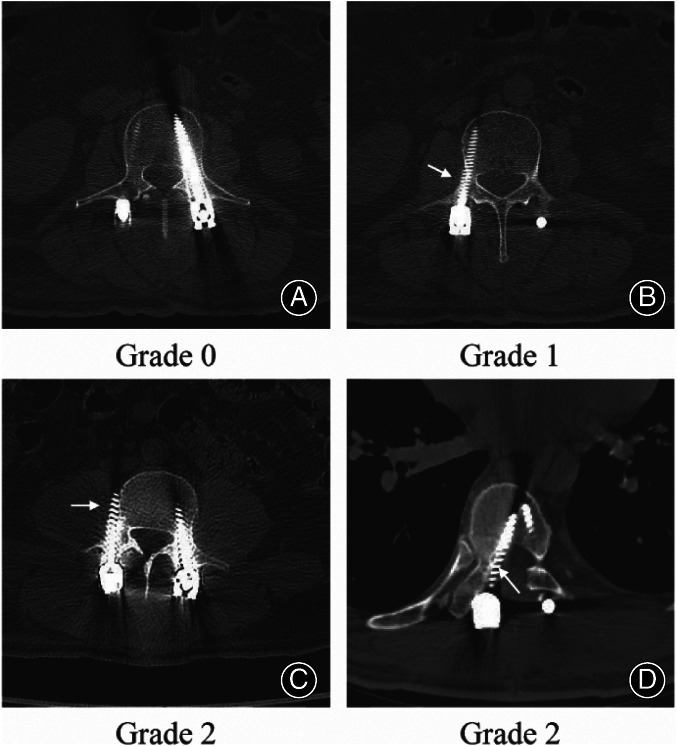
Postoperative axial images. (A) Pedicle screws of Grade 0 (screw position within the pedicle); (B) Right pedicle screw of Grade 1 (cortical breach of less than 2 mm) (arrow); (C, D) Right pedicle screws of Grade 2 (cortical breach of more than 2 mm) (arrow).

The primary objectives of this study included assessing the accuracy of pedicle screw placement and the frequency of intraoperative X‐ray usage. Accuracy of screw placement was evaluated postoperatively through CT imaging, utilizing the Gertzbein–Robbins classification^20^ and modified as follows (Figure 2): Grade 0: screw position if within the pedicle; Grade 1: cortical breach of less than 2 mm; Grade 2: cortical breach of more than 2 mm (it was thought to be dangerous to surrounding tissues). Intraoperative x‐ray usage was tallied per screw following C‐arm fluoroscopy checks, with accumulation of the number of X‐ray times were resorted to after displacement corrections.

The secondary indicator was the duration required for placing the pedicle screw, encompassing the time from the initiation of drilling a hole on the pedicle to the detachment of the tool and screw. If the screw required replacement, the duration was recorded accordingly.

### Statistical Methods

All statistical analysis was performed using SAS 9.1 software (SAS Institute, Cary, NC, USA). Results were presented as means ± SD. A paired samples *t*‐test was conducted for comparison of time for each pedicle screw placement and valid radiation exposures. *χ* ^2^‐test was used for comparison of accuracy for each pedicle screw placement. *p* < 0.05 was regarded statistically significant.

### Literature Review

We performed the search in web of science using the following search formula: (TS = [electronic OR electrical OR bioelectric OR electric) AND conductivity AND Pedicle OR Pediguard]‐Time: April 01, 2023. A total of 35 results were searched, and 21 articles were selected by removing non‐relevant studies. We selected the following key outcomes: author, year, study design, obtained accuracy of ECD, fusion levels, and number of patients/screws.

## Results

### Multicenter Randomized Clinical Trial of ECD

#### Baseline Characteristics

**FIGURE 3:**
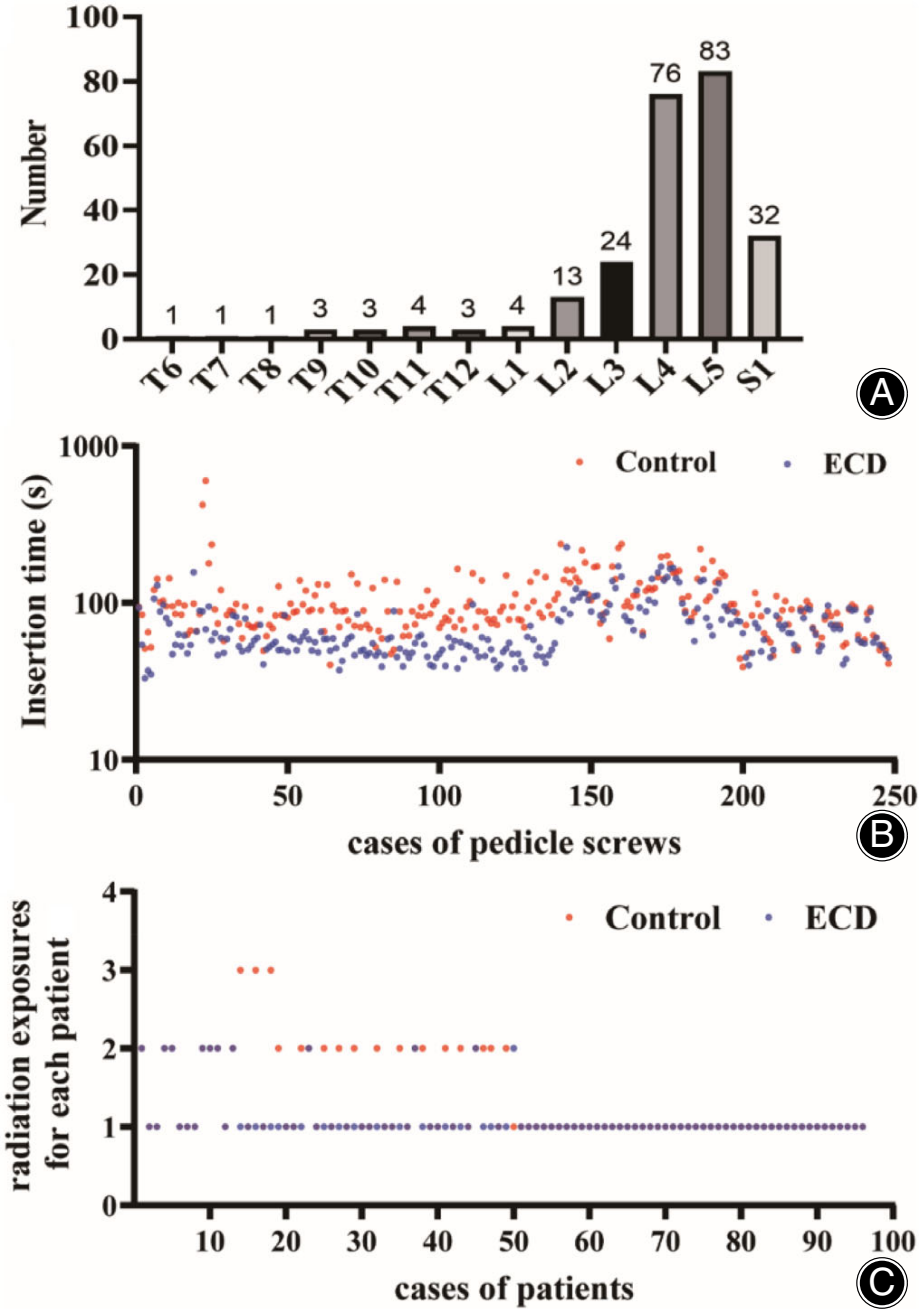
(A) Distribution of number of vertebras at each level. Screws were placed at levels from T6 to S1. Most of the screws were inserted at L4 and L5. (B) Graphs showing comparisons of insertion time for each pedicle screw. (C) Graphs showing comparisons of radiation exposures for each patient.

Regarding the baseline characteristics presented in Table 1, we enrolled a total of 96 patients, consisting of 54 males and 42 females, with an average age of 60.18 ± 11.46 years. Of those, 39 (40.63%) had lumbar disc herniation, 36 (37.50%) were diagnosed with lumbar spinal stenosis, 13 (13.54%) had spondylolisthesis, four (4.17%) suffered from thoracolumbar fracture, three (3.13%) presented with intraspinal mass, and one (1.04%) had lumbar deformity. In total, 2.58 ± 0.98 levels of vertebras were fused per patient, with 496 pedicle screws inserted at levels ranging from T6 to S1. Most of the screws were placed at L4 and L5 (Figure 3A). Neither group demonstrated any complications or unintended effects.

**TABLE 1:** Baseline demographic and surgical characteristics.

Variable	Statistic	All subjects (*N* = 96)
Age (years)	Mean ± SD	60.18 ± 11.46
	Median (Min–Max)	62.50 (18.00–86.00)
Height (cm)	Mean ± SD	164.45 ± 7.29
	Median (Min–Max)	165.00 (145.00–180.00)
Weight (kg)	Mean ± SD	67.48 ± 10.49
	Median (Min–Max)	65.50 (40.00–102.00)
Gender, *n* (%)	Male	54 (56.25%)
	Female	42 (43.75%)
Smoking, *n* (%)	Not daily	87 (90.63%)
	Daily	9 (9.38%)
Drinking alcohol, *n* (%)	Not daily	93 (96.88%)
	Daily	3 (3.13%)
Primary reason for procedure, *n* (%)	Lumbar disc herniation	39 (40.63%)
	Lumbar spinal stenosis	36 (37.50%)
	Intraspinal mass	3 (3.13%)
	Spondylolisthesis	13 (13.54%)
	Lumbar deformity	1 (1.04%)
	Thoracolumbar fracture	4 (4.17%)
Number of levels fused	Mean ± SD	2.58 ± 0.98

Abbreviation: SD, standard deviation.

#### Radiation Exposures and Inserting Time

The use of ECD has demonstrated a significant reduction in valid radiation exposures and has resulted in time‐saving benefits when conducting pedicle screw placement procedures. Within the free‐hand group, each pedicle screw placement required an average time of 101.82 ± 54.00 s and radiation exposures for each screw were at 1.30 ± 0.53 s. Conversely, within the ECD group, the average time for each pedicle screw placement was 70.88 ± 30.51 s, resulting in a notable decrease of 30.93 ± 48.44 s (*p* < 0.001), while radiation exposures for each screw were at 1.11 ± 0.32 s, showcasing a difference of 0.19 ± 0.49 that was significantly reduced (*p* < 0.001) (Table 2). These findings are well represented through the scatter plots presented in Figure 3B,C.

**TABLE 2:** Comparison of time for each pedicle screw placement and valid radiation exposures between free‐hand group and ECD group.

Variable	Free‐hand group	ECD group	Paired difference	95% CI of the difference	*p* value
Screw number	248	248			
Time (s)	101.82 ± 54.00	70.88 ± 30.51	30.93 ± 48.44	24.88–36.99	<0.001
Patient number	96	96			
Exposures times (s)	1.30 ± 0.53	1.11 ± 0.32	0.19 ± 0.49	0.09–0.29	<0.001

Abbreviation: ECD, Electronic conductivity device.

#### Accuracy and Safety

The accuracy for each pedicle screw placement between free‐hand group and ECD group is shown in Table 3. Two hundred thirty‐six (95.16%, free‐hand group) and 240 (96.77%, ECD group) screws were identified as Grade 0, four (1.61%, free‐hand group) and two (0.81%, ECD group) screws were evaluated as Grade 1, and eight (3.23%, free‐hand group) and six (2.42%, ECD group) screws were evaluated as Grade 2. There is no statistically significant difference in the accuracy of pedicle screw placement between the free‐hand group and the ECD group (*p* = 0.611).

**TABLE 3:** Comparison of accuracy for each pedicle screw placement between free‐hand group and ECD group.

Variable	Free‐hand group	ECD group	*p* value
Grade 0	236 (95.16%)	240 (96.77%)	
Grade 1	4 (1.61%)	2 (0.81%)	0.611
Grade 2	8 (3.23%)	6 (2.42%)	

Abbreviation: ECD, Electronic conductivity device.

### Literature Review of ECD

**FIGURE 4:**
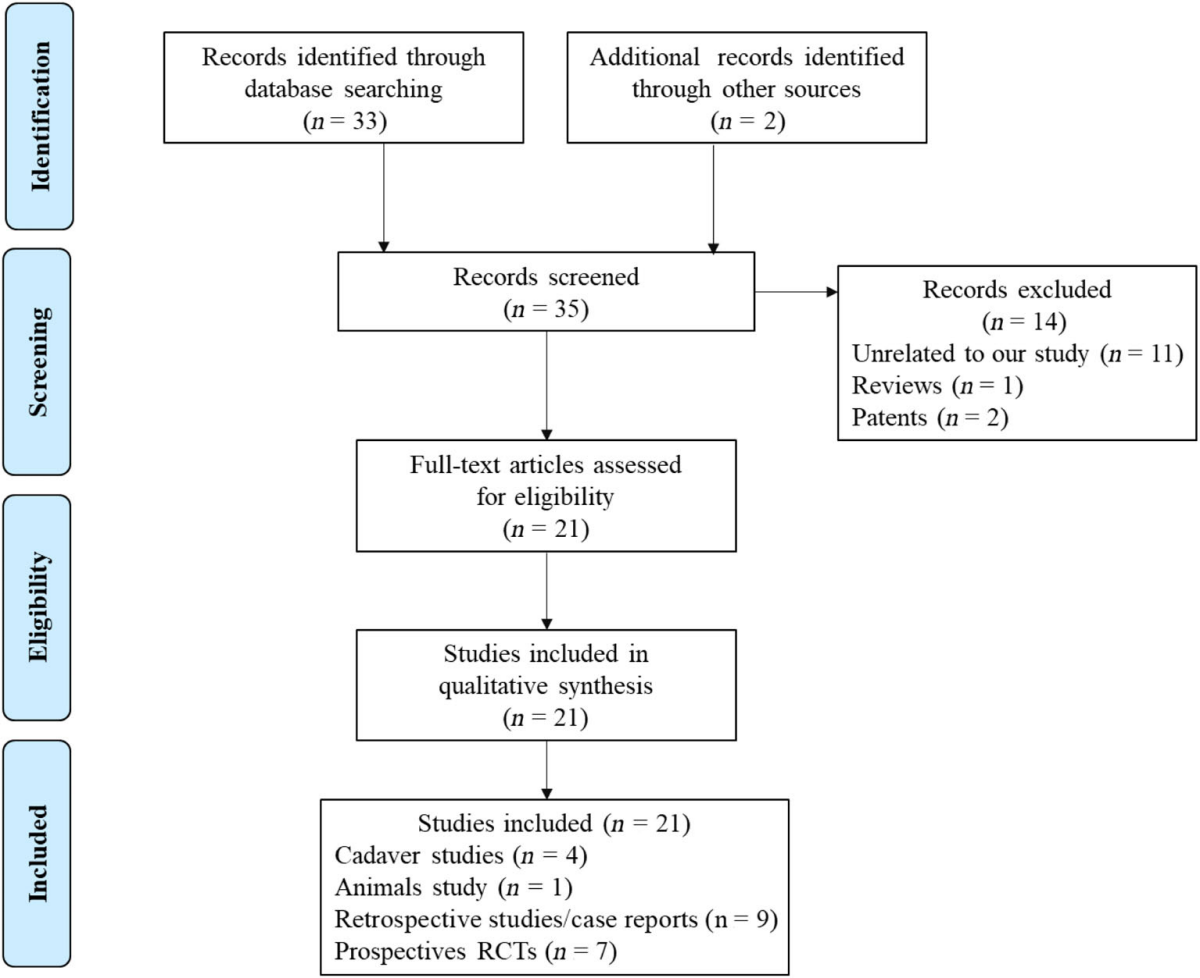
The inclusion and exclusion process of researches.

The updated literature search identified 21 studies focusing on ECD, consisting of four cadaveric studies, one animal study, nine retrospective studies, and seven prospective RCTs (Figure 4). Comprehensive outcomes of these studies are furnished in Table 4. ECD has proven to be efficacious in diverse spinal regions, including the atlas, thoracic, lumbar spine, and sacral 2‐alar‐iliac. The accuracy of ECD ranged between 85% and 100%.

**TABLE 4:** Accuracy reported for ECD.

No.	Author, year	Study design	Obtained accuracy	Spinal levels	Patients/screws
1	Bhogal *et al*., 2022^38^	Prospective randomized study	91.9%	Thoracic; Lumbar; Sacral	20 patients/114 screws
2	Yurube *et al*., 2022^32^	Retrospective case–control study	87.7%	Severe Syndromic and Neuromuscular Scoliosis (Thoracic; Lumbar)	11 patients/284 screws
3	Lebhar *et al*., 2021^37^	Prospective randomized study	85%	Thoracic; Lumbar	16 patients/78 screws
4	Kageyama *et al*., 2021^16^	Retrospective, Multi‐axis Angiography Unit + ECD	85.3% (screw position is within the pedicle);98.0% (cortical breach of less than 2 mm)	Lumbar	31 patients/150 screws
5	Kudo *et al*., 2020^33^	Retrospective	93.6%	Scoliosis (Thoracic; Lumbar)	10 patients (9 males, 1 female)/171 screws
6	Defino *et al*., 2020^36^	Prospective randomized study	97.4%	Thoracic; Lumbar	54 patients/300 screws
7	Zhai *et al*., 2019^19^	Prospective randomized clinical trial	Benefits in complicated transpedicular puncture in patients with vertebral compression >50%	Thoracic; Lumbar	61 patients (44 females, 17 males)/75 vertebras with vertebral compression fracture
8	Kageyama *et al*., 2019^39^	Technical Note, Multi‐axis Angiography Unit + ECD	87.5%	Atlas	4 patients/8 C1 lateral mass screws
9	Allaoui *et al*., 2018^31^	Retrospective Case Series	95.4%	Spinal deformities (Thoracic; Lumbar)	57 patients/882 screws
10	Dixon *et al*., 2017^28^	Cadaver	91.04%	Cervical	9 cadavers/104 drillings
11	Sandhu *et al*., 2017^40^	Retrospective	100%stable and accurate fixation	Sacral 2‐Alar‐Iliac	2 patients/4 S2‐AI screws
12	Suess *et al*., 2016^35^	Prospective randomized double‐blind comparative trials	92.8%	Thoracic; Lumbar	15 patients/84 screws
13	Guillen *et al*., 2014^14^	Cadaver	The sensitivity of the pedicle probe to detect impending breach or breach of 4 mm or less was 90.06%. The sensitivity in detecting medial wall breach was 95.8%. The positive predictive value was 87.1%.	Thoracic; Lumbar	2 cadavers/98 pedicle tracts
14	Williams *et al*., 2014^29^	Cadaver	The sensitivity to detecting cortical breach was 97% in pedicle drillings and 84% in vertebral body drillings, respectively.	Thoracic; Lumbar	2 cadavers/75 pedicle tracts
15	Bai *et al*., 2013^34^	Prospective randomized clinical trial	95.9%.ECD increases pedicle screw accuracy, especially in T1–T10.	Scoliosis (Thoracic; Lumbar)	20 patients/362 screws
16	Chaput *et al*., 2012^41^	Prospective randomized controlled trial	97.5%	Lumbar	18 patients (6 males, 12 females)/39 screws
17	Ovadia *et al*., 2011^30^	Retrospective	99.79%	Scoliosis (Thoracic; Lumbar)	98 pediatric scoliosis patients/1400 screws
18	Zeller *et al*., 2009^18^	Case series	100%.	Cervical	5 patients/26 cervical screws
19	Koller *et al*., 2009^17^	Cadaver	100% non‐critical position in anterior group, and 88.9% non‐critical position in posterior group.	Cervical; Thoracic	5 fresh‐frozen specimens/60 vertebras
20	Bolger *et al*., 2007^15^	Multi‐center clinical trial	An overall sensitivity of 98% and specificity of 99% for detecting a pedicle breach. The negative predictive value was 99.8%, with a positive predictive value of 94%.	Thoracic; Lumbar	97 patients/521 pedicle drillings
21	Bolger *et al*., 2006^13^	Experience in pigs	100% positive predictive value, 96% negative predictive value, 100% specificity, and 97% sensitivity.	Thoracic; Lumbar	11 pigs/168 screws

Abbreviation: ECD, Electronic conductivity device.

## Discussion

In this study, ECD was modified wherein six lamps of diverse colors have been incorporated on the progress bar. And then, we implemented self‐control randomization techniques in order to decrease any potential biases that could arise among both patients and vertebras. A total of 96 patients were enrolled in our trial, in which 496 pedicle screws were inserted, with 248 pairs of screws in the free‐hand group and the ECD group, respectively. The accuracy of ECD‐guided insertion was demonstrated to be 96.77%. The insertion of modified ECD was also remarkably safe, with no patients experiencing any neurovascular deficits throughout the trial. When compared with free‐hand technique, it also offered a 14.6% reduction in valid radiation exposures and a 30.38% decrease in time for pedicle screw placement. In addition, the updated literature search identified 21 studies in ECD, which showed an application in various areas of the spine such as the atlas, thoracic and lumbar spine, as well as sacral 2‐alar‐iliac, with an accuracy ranged from 85% to 100%.

### Advantages of ECD in Spinal Surgery

The precision of pedicular screw placement has been a significant consideration in spinal surgery for years,^21^ with current techniques including the surgeon's experience with mechanical feedback, navigation and robotic systems, spinal cord monitoring, and others.^22^, ^23^ These techniques offer their own benefits, and also have drawbacks such as high cost, reliance on operator skill, and the need for neurophysiologists.^24^ Additionally, surgeons face exposure to both direct and scattered ionizing radiation during procedures.^25^ The spinal surgeon's intraoperative radiation exposure may be unacceptable since they might exceed the lifetime limit in less than 10 years.^26^ And incorrectly placed screws may only become apparent later in the process with the use of C‐arm fluoroscopy.^27^ Alternatively, ECD presents a viable option for real‐time navigation without the risks associated with ionizing radiation. And we modified the ECD with six differently colored lamps on the progress bar in this study. It offers different colors and various numbers of lights to provide informative cues. As a result, we believe ECD has the advantages of affordability, portability, and real‐time detection capabilities.

### Safety and Effectiveness of the Modified ECD with Literature Review

ECD is highly promising in improving the accuracy of pedicle screw placement. This innovation not only facilitates the identification of the optimal trajectory but can also identify cortical violations prior to complete penetration. In 2006, Bolger *et al*.^13^ first reported the high reliability of impedance measurement to detect iatrogenic initial pedicle perforation in pigs. In 2007, Bolger *et al*.^15^ conducted a multi‐center clinical trial in 97 patients with 521 pedicle drillings, and reported an overall sensitivity of 98% and specificity of 99% for detecting a pedicle breach, which was twice better than the conventional technique. Since then, studies carried out to prove the accuracy in different conditions. Koller *et al*.^17^ and Dixon *et al*.^28^ did preliminary researches on cadavers on cervical and thoracic spine. Williams *et al*.^29^ also did a cadaver study and found that the sensitivity to detecting cortical breach was 97% in pedicle drillings and 84% in vertebral body drillings, respectively. Ovadia *et al*.^30^ reported a significant decrease in neuro‐monitoring alarms during scoliosis surgeries of a magnitude of three‐fold. In retrospective studies, Allaoui *et al*.,^31^ Yurube *et al*.,^32^ and Kudo *et al*.,^33^ showed the accuracy between 87.7% and 93.6%. Kageyama *et al*.^16^ showed a 98% accuracy in retrospective lumbar surgeries with ECD and multi‐axis angiography unit. For prospective randomized controlled trials, Bai *et al*.,^34^ Suess *et al*.,^35^ Defino *et al*.,^36^ Lebhar *et al*.,^37^ and Bhogal *et al*.,^38^ documented the accuracy from 85% to 97.4%, and shows that ECD increases pedicle screw accuracy, especially in scoliosis surgeries. New applications and techniques have been designed for ECD. Besides the percutaneous transpedicular puncture in patients with vertebral compression fractures,^19^ Kageyama *et al*.^39^ found ECD was useful for an optimal positioning of bi‐cortical screws in the lateral mass of C1 atlas. Sandhu *et al*.^40^ used ECD in sacro‐iliac screw and found 100% stable and accurate fixation.

Besides the improvement of accuracy rate, reduction of radiation exposure is another primary objective in this study. In the open surgery, Chaput *et al*.^41^ and Bai *et al*.^34^ reported that ECD reduced nearly 30% X‐ray shots in open surgery. Accordingly, it might reduce more X‐ray shots in minimally invasive spine surgery since Zhai *et al*.^19^ did a prospective randomized clinical trial for percutaneous transpedicular puncture in patients with vertebral compression fractures and found that ECD reduced more than 3‐times the fluoroscopy frequency in complicated transpedicular puncture in patients especially with vertebral compression >50%. In the present study, radiation exposures for each screw in the ECD group were 1.11 ± 0.32 s, with a reduction of 0.19 ± 0.49 (14.6%) (*p* < 0.001).

Importantly, ECD is also proved of saving time for pedicle screw placement. Bai *et al*.^34^ reported that ECD can save 15% surgical time during screw placement. Koller *et al*.^42^ reported that ECD speeded up the surgery of ankylosing spondylitis. It is important to shorten surgical time since it may reduce hemorrhage and make the operation safer.^43^ This study showed that time for each pedicle screw placement was 70.88 ± 30.51 s in ECD group, which decreased 30.38% during the screw insertion.

## Limitations and Strengths

This study offers convincing evidence using a prospectively randomized self‐control method that has been applied to patients. The gathered data reveals that the ECD method demonstrates a similar degree of accuracy to the standard freehand technique which relies heavily on C‐arm fluoroscopy. As a result, the ECD technique efficiently reduces the exposure to X‐rays and significantly reduces the time required to complete pedicle screw insertion.

However, this study has some limitations. It is important to note that the study was conducted by three operators each possessing over 20 years of experience in spine surgeries and did not investigate the data of junior residents. Junior surgeons may feel more comfortable utilizing navigation systems to attain a safer procedure. Furthermore, the study did not encompass the medico‐economic effects and as the ECD method has yet to be priced. Equally, the comparison exclusively refers to conventional free‐hand techniques, therefore, skipping other navigation devices such as navigation and robotic systems. Nevertheless, the ECD method is portable, affordable, and practical in comparison to larger devices. Additionally, in the future, integrating ECD with navigation or robotic systems could enable biofeedback and further improve safety during procedures.

## Conclusions

ECD is a safe technique offering reduced 14.6% valid radiation exposures and 30.38% in time for pedicle screw placement compared with free‐hand technique. The prospectively randomized trial and the review indicate that ECD are of interest for the application in combination with other techniques for a safe and automated surgical procedure.

## Author Contributions

Xiao Zhai: Conceived and designed the experiments, and wrote the paper. Bo Li: Performed the experiments and wrote the paper. Kai Chen, Ziqiang Chen, Jie Shao, and Kai Chen: Analyzed the data. Qintong Xu and Dehua Meng: Performed the experiments. Qinming Fei, Leisheng Jiang, and Yushu Bai: Performed the surgeries and critical review. Ming Li: Critical review. All authors read and approved the final manuscript.

## Ethics Statement

Shanghai Changhai Hospital Ethics Committee, CHEC2017‐179; Shanghai Zhongshan Hospital Ethics Committee, 2017‐101; Ethics Committee of Xinhua Hospital, HXEC‐B‐2017‐004‐2.

## Conflict of Interest Statement

The authors declare no conflict of interests.

## Supporting information


**Video S1.** The ECD is designed to supply real‐time sound and light signals.
